# Evaluating the Antioxidant Potential of Coumestrol in the Treatment of Tripterygium Glycoside-Induced Oligospermia in Rats and Its Potential Mechanisms

**DOI:** 10.3390/vetsci13030224

**Published:** 2026-02-26

**Authors:** Yongzheng Liu, Sikai Chen, Kang An, Long Chen, God’spower Bello-Onaghise, Yu Zhang, Shunda Li, Mo Chen, Haoran Wang, Qianwei Qu, Yanhua Li

**Affiliations:** 1 College of Veterinary Medicine, Northeast Agricultural University, 600 Changjiang Road, Xiangfang, Harbin 150030, China; s230601005@neau.edu.cn (Y.L.); s230602057@neau.edu.cn (S.C.); s230601006@neau.edu.cn (K.A.); s220602074@neau.edu.cn (L.C.); godspower.bello-onaghise@uniben.edu (G.B.-O.); s230602064@neau.edu.cn (Y.Z.); s220602070@neau.edu.cn (S.L.); 2 Department of Animal Science, Faculty of Agriculture, University of Benin, Benin City 300103, Nigeria; 3 State Key Laboratory of Veterinary Biotechnology, Harbin Veterinary Research Institute, Chinese Academy of Agricultural Sciences, Harbin 150069, China; chenmo1212@foxmail.com; 4 Department of Clinical Medicine, School of Clinical Medicine, Southern Medical University, 1023 Shatainan Road, Guangzhou 510515, China; 3200901024@i.smu.edu.cn

**Keywords:** tripterygium glucosides, oxidative stress, oligospermia, coumestrol, antioxidant, network pharmacology

## Abstract

Tripterygium glycoside (TG) is known to disrupt the oxidative balance in bio-systems, inducing oxidative stress-mediated toxic effects on testicular tissue. This study aimed to explore the therapeutic potential of coumestrol (COU) against these adverse effects. Sixty-four male Sprague–Dawley rats were randomized into control and Tripterygium glycoside (TG) groups for four weeks. Following initial intervention, eight rats per group were sacrificed to verify the establishment of the oligospermia model and hormonal dysfunction. The remaining rats were subdivided into five therapeutic subgroups, TG, TG + L-carnitine, and three COU dosage groups (low, medium, and high) to evaluate potential protective effects. The present study comprehensively analyzed its impacts on testicular histomorphology, circulating testosterone, follicle-stimulating hormone (FSH) and luteinizing hormone (LH) levels, and redox balance status, as well as a suite of serum biochemical and physiological biomarkers. Our results revealed that TG induced oligospermia in rats, causing significant testicular oxidative stress characterized by excessive accumulation of reactive oxygen species (ROS) and malondialdehyde (MDA), alongside depleted superoxide dismutase (SOD) activity and total antioxidant capacity (T-AOC). Conversely, COU treatment effectively mitigated these impairments by significantly downregulating ROS and MDA levels while restoring SOD activity and T-AOC.

## Simple Summary

Tripterygium glycoside (TG) is recognized for its therapeutic potential against autoimmune conditions, including rheumatoid arthritis, glomerulonephritis, nephrotic syndrome, and lupus erythematosus. However, TG is also known to disrupt the oxidative balance in bio-systems, inducing oxidative stress-mediated toxic effects on testicular tissue. Coumestrol (COU) has been reported to ameliorate the deleterious impacts of cryo-damaged ovine semen by improving sperm antioxidant capacity, mitochondrial activity, acrosome, and DNA integrity. However, there is still limited knowledge about its reproductive efficacy in male animals. In this study, COU effectively ameliorated the oligospermia caused by TG toxicity in the testes of the experimental rats by increasing their antioxidant capacity through the production of antioxidant enzymes and reversing the oligospermia caused by TG toxicity in the testes of the experimental animals. Combining Western blot assays with network pharmacology analysis, we predicted that TG-induced reproductive toxicity may be associated with the PI3K-AKT/MAPK signaling cascades. Notably, the analysis suggested that COU might counteract this pathway activation, thereby alleviating TG-mediated damage and ultimately enhancing sperm motility and concentration.

## 1. Introduction

Tripterygium glycoside (TG) is a bioactive extract derived from the roots of the traditional Chinese herb Tripterygium wilfordii, commonly referred to as Thunder God Vine. TG exhibits significant immunosuppressive and anti-inflammatory properties; as such, it is extensively utilized in clinical settings for the management of a diverse range of refractory autoimmune and inflammatory diseases. These include, but are not limited to, rheumatoid arthritis, nephrotic syndrome, glomerulonephritis, systemic lupus erythematosus, and thrombocytopenic purpura [1,2,3]. The broad therapeutic efficacy of TG is primarily attributed to its key active component, triptolide [4].

Despite its therapeutic value, the clinical application of TG is limited by its broad spectrum of adverse effects, particularly its toxicity to the reproductive system. While TG can affect digestive and hematological functions, its impact on male fertility is most concerning, manifesting as testicular atrophy, decreased sperm concentration, and reduced motility [5,6]. Consistently, studies in adult male animal models have confirmed that TG exposure significantly impairs spermatogenesis and induces reproductive toxicity [7,8]. Mechanistically, TG-associated testicular injury involves complex pathways, including inflammation, ferroptosis, and notably, the disruption of oxidative balance [9,10]. Such alterations in oxidative stress status, alongside metabolic changes in markers like glyceryl phosphorylcholine and citrate, are critical contributors to male infertility [11]. excessive reactive oxygen species (ROS) accumulation compromises sperm structural integrity, causing DNA strand breaks and epigenetic modifications; severe oxidative damage exceeds the self-repair capacity of spermatozoa, triggering apoptosis and diminishing semen quality [12]. Furthermore, the abundance of polyunsaturated fatty acids in the sperm plasma membrane renders it highly susceptible to ROS-induced lipid peroxidation, a key factor driving the loss of sperm motility [13].

Coumestrol (COU), a natural pterocarpan belonging to the phytoestrogen subgroup of coumestans, is widely distributed in legumes, soybeans, and traditional Chinese medicines such as *Radix puerariae* [14]. Unlike a broad range of phytochemicals, COU is particularly noted for its potent antioxidant activity and its high affinity for estrogen receptors (ERα and ERβ), which are crucial regulators of the male reproductive system [15,16]. Recent evidence suggests that COU can maintain cellular homeostasis by scavenging ROS and modulating apoptotic pathways [17]. Specifically, COU has been shown to improve sperm antioxidant capacity and mitochondrial integrity in cryo-damaged ovine semen [18]. TG are known to induce oligospermia primarily through oxidative stress-mediated damage to the blood-testis barrier and germ cell apoptosis [19,20]. COU’s ability to mitigate oxidative insults positions it as a potential therapeutic candidate. However, research regarding its efficacy in protecting against drug-induced male reproductive dysfunction remains insufficient.

Given the complex subnetworks inherent in disease pathogenesis, the traditional reductionist paradigm of “one disease, one target, one drug” is often insufficient for elucidating the efficacy of multi-component therapeutics [21]. In contrast, network pharmacology offers a systemic perspective, enabling the holistic analysis of intricate protein interactions and signaling nodes that drive biological functions [22,23]. By integrating this approach with molecular docking simulations, researchers can effectively predict ligand-protein binding affinities and screen potential bioactive candidates from existing databases. This integrated strategy is crucial for identifying key therapeutic targets and correcting dysregulated networks, ultimately facilitating the discovery of effective treatments [24,25,26].

This study established a TG-induced oligospermia model in male rats, assessed COU’s efficacy in ameliorating the condition, and applied network pharmacology to deduce the possible mechanisms underlying TG toxicity enhancement and COU’s therapeutic potential.

## 2. Materials and Methods

### 2.1. Animals Experiment

A total of 64 six-week-old Sprague–Dawley rats (weighing 200 ± 20 g) were procured from Liaoning Changsheng Biotechnology Co., Ltd. (Benxi, China) The animals were individually housed under standard laboratory conditions (22 ± 3 °C, 55 ± 5% humidity, 12 h light/dark cycle) with ad libitum access to standard chow and water. The study was approved by the Institutional Animal Care and Use Committee of Northeast Agricultural University (NEAUEC2024 03 134).

Model Establishment and Verification: After a one-week acclimation following ARRIVE guidelines [27], the 64 rats were initially randomized into two cohorts: the Control group (*n* = 16) and the Model group (*n* = 48). The Model group was administered 40 mg/kg Tripterygium glycosides (TG) suspension daily via gavage for four weeks. The Control group received an equivalent volume of deionized water (10 mL/kg). Immediately following the four-week induction period (Day 28), 8 rats from the Control group and 8 rats from the Model group were randomly selected and sacrificed to verify the model. Blood samples and epididymal tissues were collected. The model was considered successfully established based on the criteria of a statistically significant reduction (*p* < 0.05) in sperm density, sperm motility, and serum testosterone levels in the TG-treated rats compared to the controls.

Grouping and Treatment: The remaining 48 rats (8 from the original Control group and 40 from the Model group) entered the treatment phase. The 8 remaining Control rats continued as the Control group. The 40 Model rats were further randomized into five subgroups (*n* = 8 per group): Model group (TG), Positive Control group (TG + LC): a standard therapeutic agent for male infertility used as a positive control [28,29], Low-dose COU group (TG + COU-L), Medium-dose COU group (TG + COU-M), High-dose COU group (TG + COU-H). The Control group and TG group received an equivalent volume of deionized water. The rats were randomized into different groups using a computer-generated random number sequence to ensure unbiased allocation.

### 2.2. TG Dosage Preparation and Administration

TG tablets, each containing 10 mg, were procured from Yuanda Pharmaceutical (Batch No.: 20230801, Huangshi, China) and formulated into a 5 mg/mL suspension using saline. Rats were administered 1 mL per 0.1 kg of body weight of this suspension daily via gavage, ensuring a consistent dosage of 40 mg/kg of body weight. Dosages were adjusted according to weight changes to maintain accuracy, in accordance with established research standards [30,31].

### 2.3. Administering and Preparing Dosages of COU and Levo-Carnitine (L-C)

The source of COU was MedChem Express Co., Ltd. (S148519, Shanghai, China), and L-C came from Northeast Pharmaceutical Group Shenyang First Pharmaceutical Co., Ltd. (231004, Shenyang, China). The body weights of the rats were recorded weekly prior to drug administration. Deionized water was administered to the control and model groups at 10 mL/kg/day. The TG + L-C group received 0.2 g/kg/day of L-carnitine. The TG + COU-L, TG + COU-M, and TG + COU-H groups were given 20 µg/kg, 30 µg/kg, and 40 µg/kg of COU, respectively. Each treatment was given through gavage at a rate of 1 mL per 0.1 kg of body weight daily for 4 weeks [31].

### 2.4. Collection and Processing of Blood and Tissue Samples

After 4 weeks of treatment, rats were fasted for 12 h (with free access to water) before sample collection. Blood was drawn from the abdominal aorta under anesthesia (1% pentobarbital sodium, 50 mg/kg ip), transferred to heparinized tubes, and then centrifuged at 3000 rpm for 20 min at 4 °C to separate serum. The harvested serum samples were preserved at −80 °C for subsequent biochemical analyses and hormonal detections. Subsequent to blood collection, rats were euthanized, and the left testis, epididymis, liver, and kidney were quickly removed. Tissues were rinsed with pre-cooled physiological saline, dried with filter paper, and processed as follows: Testicular tissues were fixed, embedded, sectioned, and stained with Hematoxylin and Eosin (H&E) for histological examination. Spermatogenic function was quantitatively assessed using the Johnsen score system [32], and the rest was snap-frozen in liquid nitrogen and stored at −80 °C for oxidative stress and Western blot analyses. To ensure the objectivity of the results, a single-blind study design was implemented. All samples, including serum, testicular tissues, and epididymal fluid, were labeled with unique coded identifiers. Consequently, the investigators performing the CASA, histological examination, and biochemical assays were blinded to the experimental grouping and treatment conditions. The codes were revealed only after the data collection was completed.

### 2.5. Serum Biochemical and Hormonal Analyses

Collected blood samples were incubated at 25 °C for 2 h to allow for clotting. Subsequently, the samples were centrifuged at 1000 rpm for 20 min to obtain serum. ELISA kits were used to measure the serum levels of T (Shanghai Enzyme-linked Biotechnology Co., Ltd. Cat: ml002865, Shanghai, China), LH (Shanghai Enzyme-linked Biotechnology Co., Ltd. Cat: ml002860, Shanghai, China), and FSH (Shanghai Enzyme-linked Biotechnology Co., Ltd. Cat: ml002872, Shanghai, China). All ELISA assays were performed in full accordance with the respective manufacturers’ instructions, and absorbance was quantified at a single 450 nm wavelength using an ELISA microplate reader (Hangzhou Bio-gener Technology Co., Ltd., Hangzhou, China). In addition to hormonal analysis, serum samples were evaluated for markers of hepatic and renal function, including CRE, BUN, ALT, BUN, and AST. All biochemical measurements were conducted in strict compliance with the standard protocols stipulated by the respective assay kit manufacturers.

### 2.6. Measurement of Sex Hormones Within the Testicles

To evaluate intra-testicular concentrations of the hormones T, FSH, and LH, a method from reference [33] was used. A 0.2 g testicular tissue sample was homogenized with 0.5 mL of PBS, centrifuged at 3000 rpm for 10 min, and the supernatant’s protein was measured using a BCA Protein Assay Kit. Hormone levels were then quantified with specific detection kits: testosterone with the T kit, and FSH and LH with their respective kits.

### 2.7. Evaluation of Sperm Quality

The epididymal tail dissected from each rat was transferred into a 5 mL Eppendorf tube containing 3 mL of physiological saline pre-warmed to 37 °C. The tissue was finely minced using ophthalmic scissors inside the tube, and the sample was incubated in a 37 °C water bath for 5 min to fully disperse sperm into the suspension. Subsequently, a 10 µL sample of the resulting suspension was subsequently loaded onto a glass slide, and sperm concentration and total motility (defined as the percentage of spermatozoa showing any form of movement) were analyzed automatically via a computer-assisted sperm analysis (CASA) system (WLJY-9000, Nanjing Weili Apparatus Co., Ltd., Nanjing, China). For each sperm suspension sample, the standard settings for rat sperm analysis were applied: Image acquisition rate was 60 Hz; Temperature was maintained at 37 °C; and Standard field duration was 0.5 s. The primary endpoints for assessment were Sperm Concentration (10^8^/mL) and Total Motility (%), which served as the core indicators for establishing the oligospermia model.

### 2.8. Assessment of Oxidative Stress Levels in Testicular Tissue

The residual testicular tissue was collected, minced into small fragments, and subsequently homogenized by grinding. The levels of T-AOC (Nanjing Jiancheng, Cat: A015-2-1, Unit: μmol/mg prot, Nanjing, China), SOD (Nanjing Jiancheng, Cat: A001-3-2, Unit: U/mg prot, Nanjing, China), MDA (Nanjing Jiancheng, Cat: A003-4-1, Unit: nmol/mg prot, Nanjing, China), and ROS (Nanjing Jiancheng, Cat: E004-1-1, Unit: RFU, Nanjing, China) in the testicular tissue were determined strictly in accordance with the operational protocols provided by the assay kit manufacturers. All detections were performed per the manufacturer’s instructions, and testicular tissue homogenate protein concentration was standardized via BCA assay (Nanjing Jiancheng, Cat: A045-4-2, Nanjing, China) to eliminate tissue concentration interference.

### 2.9. Identifying Potential Mechanisms via Network Pharmacology

Network pharmacology was utilized to explore TG’s role in oligospermia. Disease Targets for Oligospermia: Potential targets associated with male infertility diseases (including oligospermia, azoospermia, asthenospermia) were retrieved from the DisGeNET, Malacards, and OMIM databases (searched on 20 December 2024). Genes were included only if they met the minimum bioinformatics thresholds for being considered disease-related: a disease association score ≥ 0.5 (DisGeNET) or a relevance score ≥ 2 (Malacards). Pseudogenes, non-coding RNAs, genes with only predicted (non-experimental) associations, and genes lacking clear functional annotation in UniProt were excluded.

Drug Targets for COU: Potential protein targets of COU (CAS No.: 479-13-0) were predicted using the SwissTargetPrediction and STITCH databases. To ensure high confidence, only targets with a prediction score ≥ 0.7 (SwissTargetPrediction) or an interaction confidence score ≥ 0.8 (STITCH) were retained. Proteins without human/rat orthologs (per NCBI HomoloGene), those supported only by in silico prediction without experimental binding data, and non-mammalian-specific proteins were excluded.

Network Construction and Analysis:Common genes between the screened oligospermia disease targets and COU drug targets were identified using Venny 2.1. Only genes with experimental validation in both sources were retained for further analysis. These common targets were uploaded to the STRING database to construct a protein–protein interaction (PPI) network, which was then downloaded and imported into Cytoscape software (version 3.10.2) for visualization and analysis. Key hub genes within the network were identified using the CytoHubba plugin.

Functional Enrichment: Gene Ontology (GO) and Kyoto Encyclopedia of Genes and Genomes (KEGG) pathway enrichment analyses for the common targets were performed using ShinyGO 0.81 to elucidate the involved biological processes, cellular components, molecular functions, and key signaling pathways.

### 2.10. Western Blotting Analysis

Using a lysis buffer with PMSF, protein was extracted from the testicular tissues of rats. Concentrations were measured via a BCA assay. Equal protein amounts underwent SDS-PAGE and were transferred to PVDF membranes. After blocking with 5% non-fat milk, membranes were incubated overnight at 4 °C with primary antibodies against AKT1 (Cell Signaling Technology (CST), Catalog No.: 2938, Dilution: 1:1000, Danvers, MA, USA), PI3K (Cell Signaling Technology (CST), Catalog No.: 4249, Dilution: 1:1000, Danvers, MA, USA), ERK1/2 (Cell Signaling Technology (CST), Catalog No.: 4695, Dilution: 1:1000, Danvers, MA, USA), and GAPDH (Abcam, Catalog No.: ab9485, Dilution: 1:5000, Cambridge, UK). Afterward, they were exposed to HRP-linked secondary antibodies (Beyotime Biotechnology, Catalog No.: A0208, Dilution: 1:3000, Shanghai, China) for 2 h. Detection was performed using an enhanced chemiluminescence kit (Beyotime Biotechnology, Catalog No.: P0018M, Shanghai, China) and a Tanon 5200 system (Tanon Science & Technology Co., Ltd., Shanghai, China). The gray values of the protein bands were quantified using ImageJ software (ImageJ, version 1.52, NIH, Bethesda, MD, USA). The relative protein expression levels were normalized to the internal control GAPDH (Target Protein/GAPDH).

### 2.11. Data Analysis

Data analysis was performed with a one-way ANOVA using GraphPad Prism 9.5.1, and Tukey’s HSD test was used for multiple mean comparisons. Results are indicated as mean ± standard deviation (X ± SD), with statistical significance thresholds at * *p* < 0.05, ** *p* < 0.01, *** *p* < 0.001, and ns, not significant.

## 3. Results

### 3.1. COU Attenuated TG-Induced Body Weight Loss in Rats

**Figure 1:**
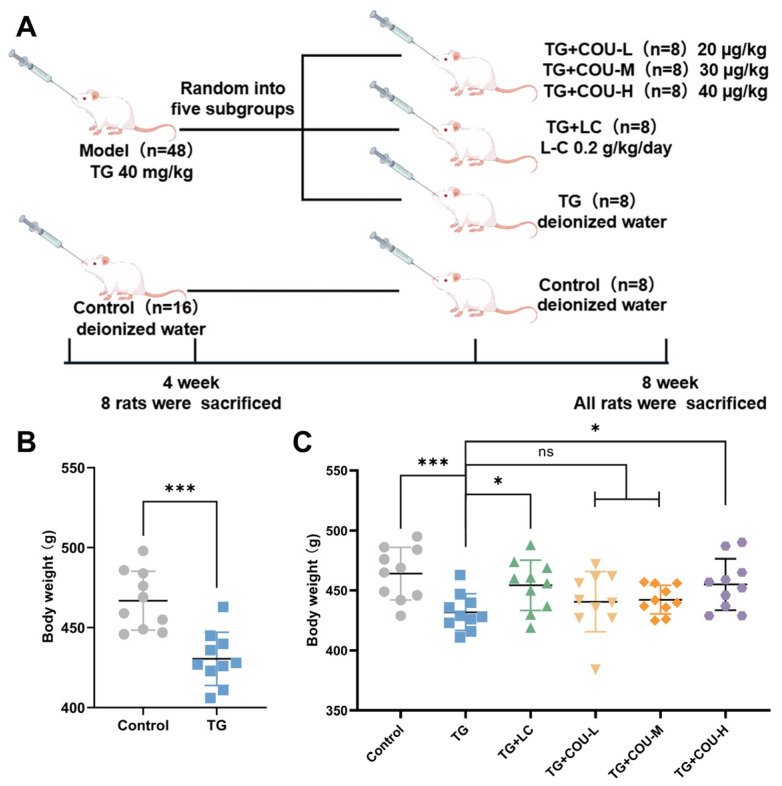
Effects of TG and COU on rat body weight of rats. (**A**) Animal Experiment Flow Chart. (**B**) TG significantly (*p* < 0.001) reduced body weight (g). (**C**) COU significantly mitigated the effect of TG on body weight at high concentration (*p* < 0.05).

TG significantly reduced the body weights of rats compared with the control group in the experiment (*p* < 0.01). Rats in the TG + LC, TG + COU-L and TG + COU-M groups did not show significant differences in body weight compared to the TG group (*p* > 0.05). However, the body weight of rats in the TG + COU-H group was significantly higher than that of the TG group (*p* < 0.05) (Figure 1A,B).

### 3.2. Sperm Quality Is Improved by COU

**Figure 2:**
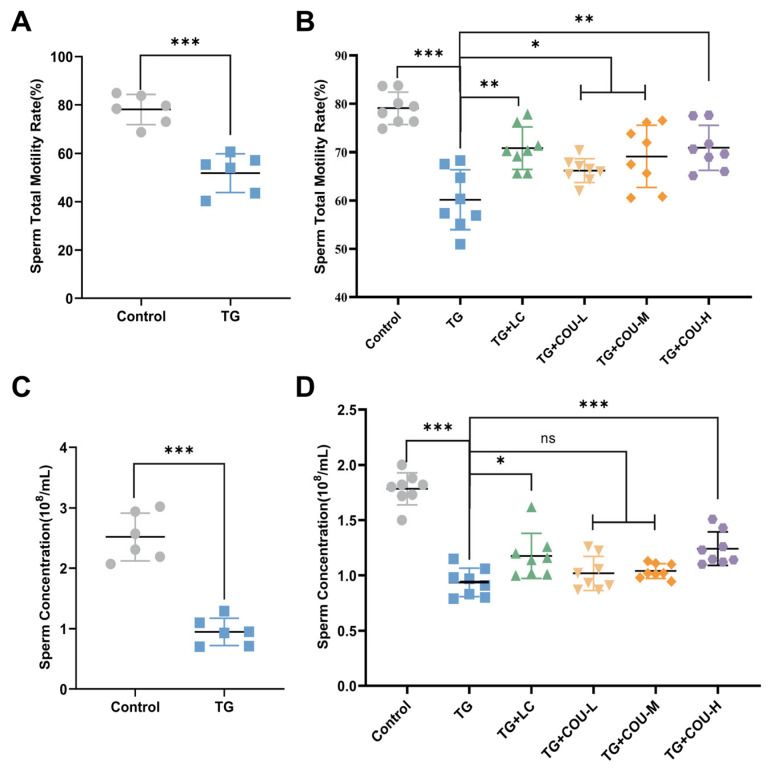
Impact of TG and COU on sperm parameters. (**A**) Impact of TG on Sperm Total Motility Rate. (TG vs. Control, *p* < 0.001). (**B**) Impact of TG on Sperm Concentration. (TG vs. Control, *p* < 0.001). (**C**) Improvement of sperm Total Motility by COU. Compared to TG, COU at low and medium (*p* < 0.05) and High (*p* < 0.01) concentrations significantly increased the reduced level of motility. (**D**) Improvement of sperm concentration by COU. Compared to the TG group, COU significantly ameliorated the toxicity of TG at high concentrations in the TG + COU-H group (*p* < 0.001) but the ameliorative effect of COU was not significant in the TG + COU-M and TG + COU-L groups, even though it exhibited positive trends.

The findings showed that administering TG at a dose of 40 mg/kg/d for four weeks via gavage markedly impaired sperm quality, compared with the control group, the model group exhibited a significant reduction in sperm motility and concentration. (*p* < 0.001; Figure 2A,B).

Regarding sperm motility, significant progressive improvements were observed: TG + COU-L vs. TG (*p* < 0.05), TG + COU-M vs. TG (*p* < 0.01) and TG + COU-H vs. TG (*p* < 0.001) (Figure 2C). As for sperm concentration, a significant increase was only detected in the high-dose COU group (TG + COU-H) when compared to the TG group (*p* < 0.01), while no significant differences were found for the low- or medium-dose groups (*p* > 0.05; Figure 2D).

### 3.3. COU Ameliorates Hormonal Imbalance

**Figure 3:**
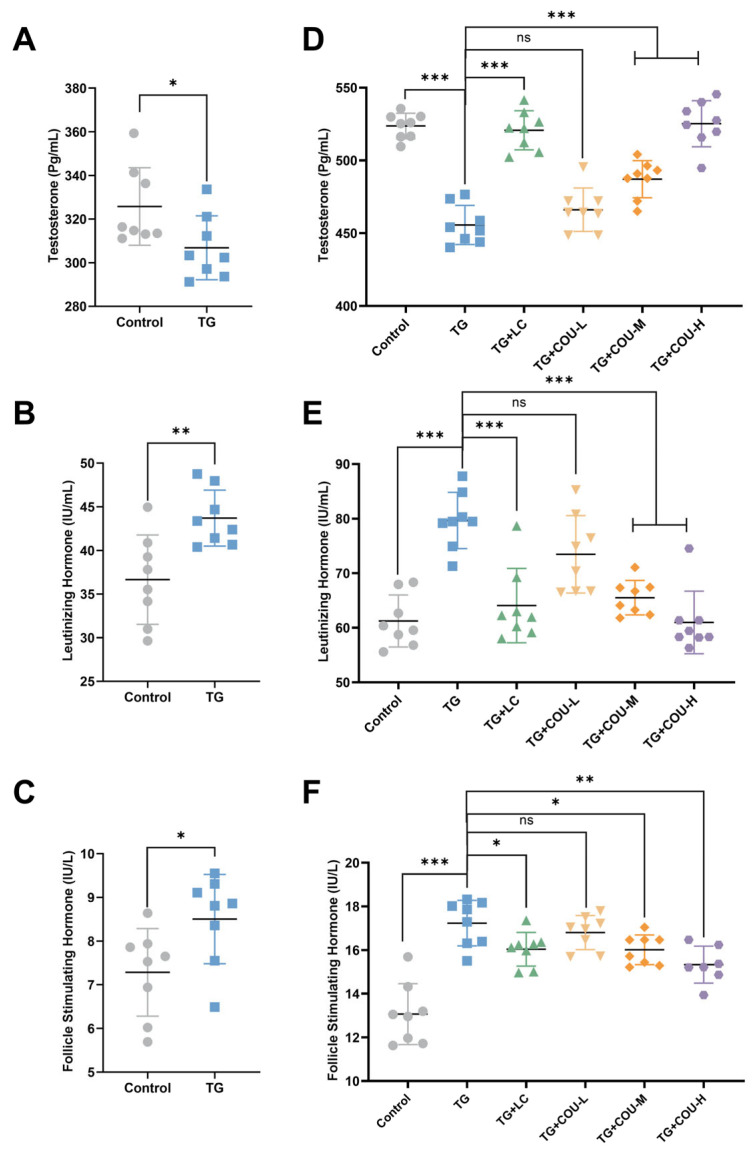
COU significantly increased testosterone levels and reduced the increased levels of LH, and FSH to restore normal function. (**A**) Alterations in serum T levels (pg/mL) following TG-induced modeling. (**B**) Impact of TG treatment on LH concentrations (IU/mL). (**C**) Changes in FSH levels (IU/L) in the TG model group. (**D**) Compared with the TG group, COU significantly increased the TG-induced reduction in Testosterone levels in the TG + COU-M (*p* < 0.001) and the TG + COU-H (*p* < 0.001). (**E**) Compared with the TG group, COU significantly reduced the increase in LH levels in the TG + COU-M (*p* < 0.001) and in the TG + COU-H (*p* < 0.001) groups. Levocarnitine (TG + LC) produced a significant increase in serum T and a decrease in LH levels (*p* < 0.001). (**F**) Compared with the TG group, COU significantly reduced the increased levels of FSH in the TG + COU-M (*p* < 0.05) and TG + COU-H group (*p* < 0.01). The ameliorative effect of COU on FSH was more potent than that of Levocarnitine (TG + LC) (*p* < 0.05).

The results revealed that T levels decreased significantly (*p* < 0.05) while LH and FSH increased significantly (*p* < 0.01 for LH and *p* < 0.05 for FSH) in the TG (model) group compared to the control group (Figure 3A–C). Following treatment with COU, there was a significant increase in the TG-induced reduction in T levels in the TG + COU-M (*p* < 0.001) and TG + COU-H (*p* < 0.001) groups compared with the TG group. Notably, there was no significant difference in T levels among the control, TG + LC, and TG + COU-H groups (Figure 3D).

The increased levels of LH and FSH in the TG group were also significantly reduced by COU. At medium and high concentrations, COU significantly reduced the elevated levels of LH (*p* < 0.001) compared to the TG group. A similar result was observed in the TG + LC group, as levocarnitine (TG + LC) also significantly reduced LH (*p* < 0.001) compared to the TG group (Figure 3E). Furthermore, the elevated levels of FSH were also significantly (*p* < 0.05 and *p* < 0.01) reduced by COU at medium and high concentrations compared with the TG group (Figure 3F). Significant effect was also observed for Levocarnitine (TG + LC) (*p* < 0.05). This further confirms that the efficacy of COU in mitigating the toxicity of TG.

### 3.4. COU Enhances Antioxidant Ability and Reduces Oxidative Imbalance in Rat Testes

**Figure 4:**
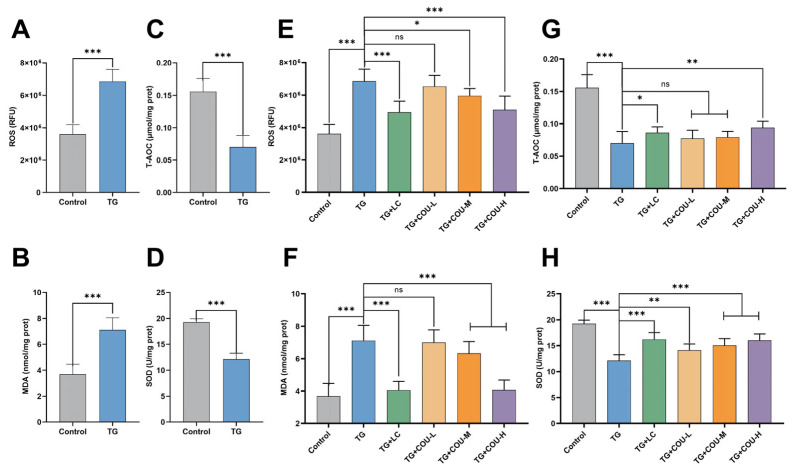
Assessment of testicular antioxidant defense system in response to COU treatment over a 4-week duration. (**A**) Effect of TG on ROS (RFU). (**B**) Effect of TG on MDA level (nmol/mg prot). (**C**) Effect of TG on T-AOC (µmol/mg prot). (**D**) Effect of TG on SOD (U/mg prot). (**E**) Effect of COU treatment on ROS (TG vs. TG + COU-H, *p* < 0.001). (**F**) Effect of COU treatment on MDA levels (TG vs. TG + COU-H, *p* < 0.001). (**G**) Effect of COU treatment on T-AOC (TG vs. TG + COU-H, *p* < 0.01). (**H**) Effect of COU treatment on SOD levels [TG vs. TG + COU-L (*p* < 0.01), TG vs. TG + COU-M (*p* < 0.001), & TG vs. TG + COU-H, (*p* < 0.001)]. Additionally, LC also significantly improved the antioxidant status of rats compared to the model (TG) group [(ROS:T C vs. TG, *p* < 0.001); (MDA: TG + LC vs. TG, *p* < 0.001); (T-AOC:TG + LC vs. TG, *p* < 0.05); (SOD:TG + LC vs. TG, *p* < 0.001)].

Based on the literature, we hypothesized that the reduction in sperm quality and the hormonal dysfunction of serum sex hormones and intra-testicular testosterone (ITT) and FSH in the TG groups were due to increased oxidative stress conditions. Thus, to assess the testicular antioxidant capacity of the testicular tissues of the animals, we measured the levels of ROS, MDA, T-AOC and SOD, which are markers of oxidative stress. Compared with the control group, the levels of ROS and MDA were significantly greater, significant reductions in T-AOC and SOD levels were observed in the TG group (*p* < 0.001) (Figure 4A–D).

With the treatment of COU, there were significant enhancements in antioxidant status in both the TG + COU-H and TG + LC groups, with reduced ROS and MDA (*p* < 0.001) and increased T-AOC levels (*p* < 0.001 for TG + COU-H and *p* < 0.05 for TG + LC). SOD levels also improved in TG + COU-L (*p* < 0.01), TG + COU-M (*p* < 0.001), and TG + COU-H (*p* < 0.001) groups (Figure 4E–H). Thus COU decreased oxidative stress by boosting the antioxidant levels in the testicular tissues derived from the treated rat group.

### 3.5. Biochemical and Physiological Parameters

We evaluated the toxicity of TG and the effectiveness of COU on liver and kidney functions in the test rats by measuring liver ALT and AST levels, along with kidney BUN and CRE. The results showed that the levels of ALT, AST, BUN, and CRE were significantly increased (*p* < 0.05) in the TG group compared to the control group, indicating liver injury and impaired kidney function. Administration of COU notably diminished the high levels of CRE, BUN, ALT, AST, and ALT (*p* < 0.01) when compared to the TG group, indicating an improvement in kidney and liver functions.

The lowest ALT levels were observed in the TG + COU-H group, while the TG + COU-M group exhibited the lowest levels of AST, BUN, and CRE compared to the TG group. Furthermore, no significant difference was observed in AST levels between the COU-L and TG groups, furthermore, no significant difference was observed in AST levels between the TG + COU-L and TG groups (*p* > 0.05). Only the medium and high-dose groups showed statistically significant reductions compared to the TG group (Table 1).

### 3.6. Evaluation of Testicular Tissues via Histopathological Analysis

**Figure 5:**
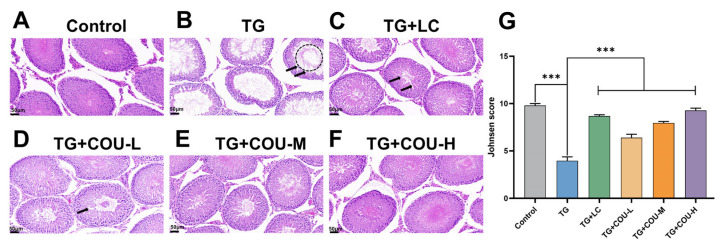
COU treatment ameliorates TG-induced testicular damage in male rats (H&E staining; scale bar = 50 µm). (**A**) HE staining of testicular tissue in the Control group. The testicular architecture appeared normal, characterized by well-arranged seminiferous tubules with intact epithelium, distinct interstitial connective tissue, and a clearly defined tubular lumen. Leydig cells and Sertoli cells were present in their typical locations and morphology; (**B**) HE staining of testicular tissue in the TG group. TG group, showing damaged tissues with disorganized and vacuolated seminiferous tubules (arrow heads), large intertubular spaces (area under dotted line) with gaps between seminiferous tubules, and desquamated cells (DC) (arrows) consistent with oligospermia; (**C**) HE staining of testicular tissue in the TG + LC group. TG + LC, also showing a fairly seperated arrangement of tubules (broken hexagon) and vacuolated seminiferous tubules (arrow heads); (**D**) HE staining of testicular tissue in the TG + COU-L group. TG + COU-L showing partially arranged and disorganized seminiferous tubules with some vacuolated seminiferous tubules (arrow heads), and gaps between seminiferous tubules; (**E**) HE staining of testicular tissue in the TG + COU-M group. TG + COU-M showing seminiferous tubules were regularly arranged with constricted intertubular spaces and maintained inter-tubule gaps; (**F**) HE staining of testicular tissue in the TG + COU-H group. TG + COU-H showing tubules were neatly aligned with diminished interstitial spaces and no evidence of desquamated cells (DC). (**G**) The Johnsen scores of different groups. TG-induced model group showed a significant reduction in the mean Johnsen score (*p* < 0.001), COU and LC treatment showed a significant increase (*p* < 0.001).

For the purpose of histological examination (Figure 5A–F), the control group’s testicular tissue displayed a normal arrangement of spermatogenic cells in the seminiferous tubules, without any histopathological lesions. Conversely, the TG group showed notable histopathological damage in the testicular tissue, marked by disordered and vacuolated seminiferous tubules, along with large intertubular spaces and gaps between the seminiferous tubules. COU treatment attenuated the histopathological damage to the testicular tissue, with the most improvement observed in the TG + COU-M and TG + COU-H groups compared with the TG group. The Johnsen scores provided objective, numerical confirmation of our histological observations. The TG-induced model group showed a significant reduction in the mean Johnsen score (*p* < 0.001), which was improved by COU treatment, with the high-dose COU group showing a significant recovery towards a near-normal score profile (*p* < 0.001). This data strongly supports our conclusion that COU protects testicular structure and promotes the recovery of spermatogenesis (Figure 5G).

### 3.7. COU Ameliorates Intra-Testicular Hormonal Concentrations

Given that intratesticular testosterone (ITT) is the primary driver of spermatogenesis and often mirrors circulating hormone trends, we further analyzed ITT levels to confirm whether the observed serum hormonal dysfunction reflected changes in the testicular microenvironment.

**Figure 6:**
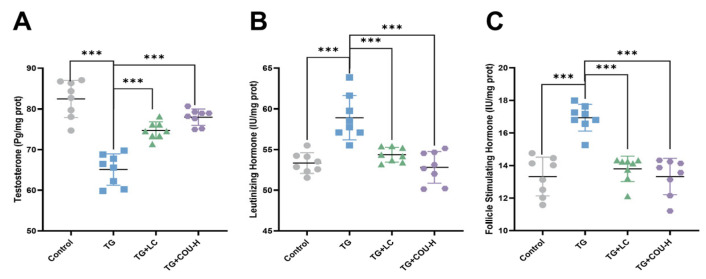
COU treatment restores intra-testicular hormone levels in TG-exposed rats. (**A**) COU (at high concentration TG + COU-H) significantly decreased testosterone (compared to the TG, *p* < 0.001). (**B**) COU (at high concentration) significantly reduced the LH level (compared to the TG, *p* < 0.001). (**C**) COU (at high concentration TG + COU-H) significantly reduced FSH level (compared to the TG, *p* < 0.001).

The results of the intra-testicular analysis showed that TG significantly reduced the intra-testicular T levels in rats (Control vs. TG) (*p* < 0.001). In contrast, COU (at high concentration in the TG + COU-H group) significantly increased intra-testicular testosterone compared with the TG group (*p* < 0.001) (Figure 6A). TG also caused significant dysregulation of intra-testicular LH. Meanwhile, at high (TG + COU-H) concentrations, COU significantly reduced the increased LH levels compared with the TG group (Figure 6B). Furthermore, significant change was observed in the intratesticular FSH levels of the experimental animals. Observation revealed that TG resulted in a notable increase in the intra-testicular FSH levels, and COU (at high concentrations) reduced this increase (Figure 6C). These changes suggest an ameliorative effect of COU on intra-testicular T, LH, and FSH levels.

### 3.8. Potential Mechanisms Through Network Pharmacology

**Figure 7:**
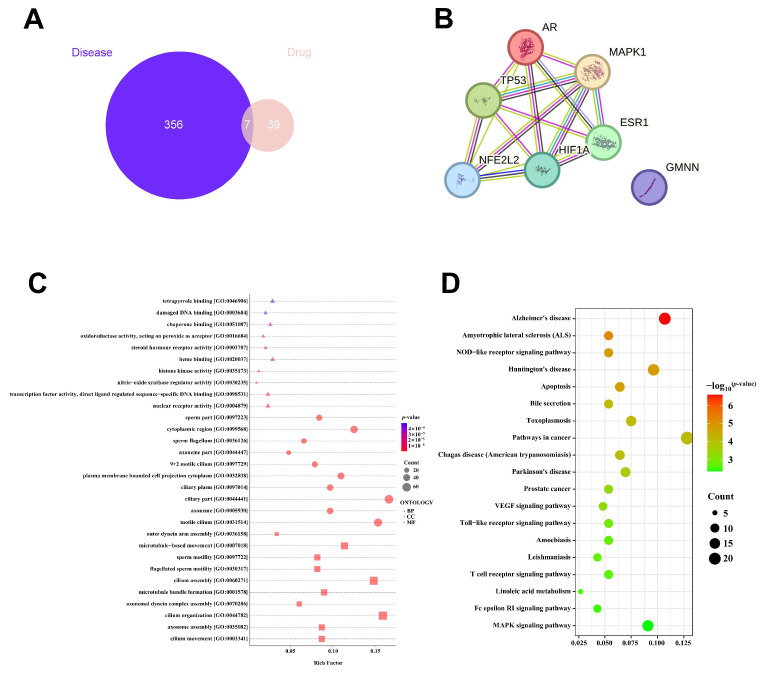
Prediction of Mechanism and signaling pathways involved in TG toxicity of and the anti-oxidant effect of COU in experimental. (**A**) Diagram of Venn showing the forecasted target genes for both Oligospermia and COU. (**B**) Cytoscape prediction of top ten hub genes of the Oligospermia PPI network. (**C**) Analysis of Biological Processes through GO Enrichment. (**D**) Analysis of KEGG pathways.

**Figure A1:**
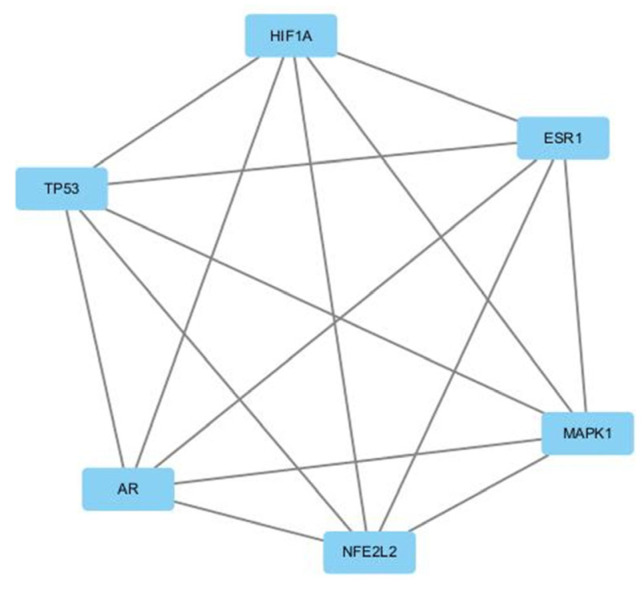
The identified 6 hub genes.

Network pharmacology analysis was performed to identify potential targets associated with oligospermia and COU. Overall, 356 targets were linked to oligospermia, and 39 targets were identified for COU. Notably, 7 targets were found to be common between the two groups (Figure 7A). These common targets were uploaded to the STRING database to generate a protein–protein interaction (PPI) network. The PPI network analysis for oligospermia revealed ten hub genes, including AR, MAPK1, TP53, NFE2L2, HIF1A, ESR1 and GMNN (Figure 7B). The PPI network was then exported as a CSV file and imported into Cytoscape 3.10.2 software for further analysis. Within Cytoscape, the CytoHubba plugin was employed to identify hub genes using the degree centrality (DC) ranking method. This method facilitated the ranking of the top 6 putative hub genes potentially relevant to both COU and oligospermia (Table 2). The UniProt database validated the identified hub genes, further establishing the interaction network (Figure A1).

Enrichment analysis of the top ten modules associated with these hub genes suggested that TG potentially induces reproductive toxicity via different GO terms and KEGG pathways. Particularly, the analysis predicted that TG might interact with biological processes (BP) related to sperm concentration and motility, specifically within hormone response pathways. Additional biological processes impacted by TG included negative regulation of transcription by RNA polymerase, morphogenesis of branching structures, inhibition of cytokine production, and the suppression of epithelial cell development (Figure 7C,D).

**Figure A2:**
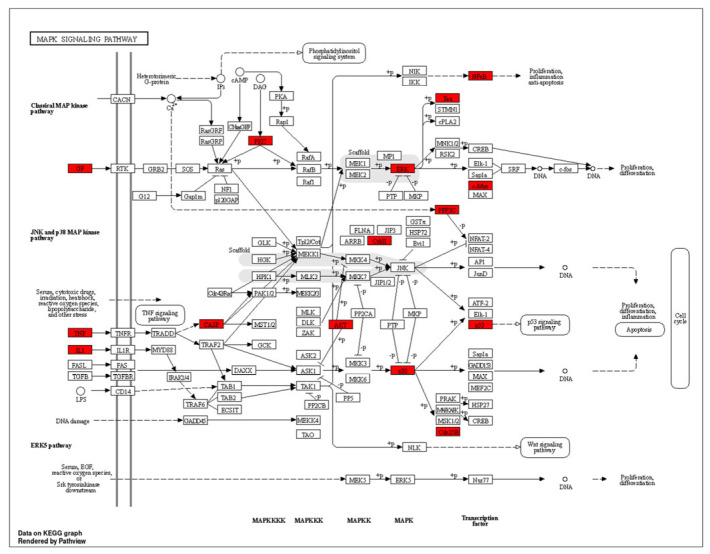
The MAPK KEGG Pathway.

These predicted dysfunctions may contribute to damage to the raft of the side membrane in the microdomain of the sperm cellular components, thereby potentially inhibiting molecular functions such as tumor necrosis factor receptor binding, tumor necrosis receptor superfamily binding, and protease binding functions exhibited by healthy sperm cells. Additionally, the KEGG pathway analysis implicated several significant pathways in the oligospermia model of the experimental rats. Among the first 50 significant pathways associated with the oligospermia model, the apoptosis, TNF signaling, and PI3K/AKT/MAPK pathways were selected for further exploration because they are closely related to oxidative stress, spermatogenesis, and cellular life cycle regulation (Figure A2).

These findings provide predictive insights into the potential mechanisms of reproductive toxicity associated with TG toxicity and the antioxidant ameliorative effect of COU. Through these aforementioned pathways and mechanisms, The computational analysis implies that COU may counteract the oxidative stress milieu created by TG administration and potentially boost the antioxidant potential of the testicular tissues in rats by modulating oxidative stress and regulating the activities of several anti-apoptotic genes.

### 3.9. ERK1/2, PI3K, and AKT Expression Is Upregulated by COU

**Figure 8:**
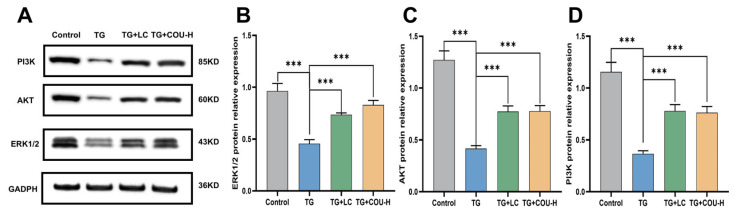
Impact of COU on PI3K, AKT, and ERK1/2 expression in test animals. (**A**) Western blot bands showing the protein expression of PI3K, AKT, ERK1/2, and the internal control GAPDH. (**B**–**D**) Quantitative analysis of relative protein expression normalized to GAPDH. COU treatment (40 µg/kg) resulted in a statistically significant upregulation of PI3K, AKT, and ERK1/2 compared to the TG group (*p* < 0.001) (the original western blot pictures can be found in Figure S1).

We selected the high-dose group (40 µg/kg) for Western blot verification, as this dose showed the most optimal therapeutic effect in phenotypic assays. The quantitative analysis, normalized to GAPDH, demonstrated that TG treatment significantly downregulated the expression of ERK1/2, PI3K, and AKT1 (*p* < 0.001) compared to the control. However, administering 40 µg/kg of COU significantly reversed this trend, leading to a statistically significant upregulation of ERK1/2, PI3K, and AKT1 (*p* < 0.001) compared to the TG group (Figure 8A–D).

## 4. Discussion

Tripterygium glycoside (TG), a compound derived from Tripterygium wilfordii, is employed in the treatment of rheumatoid arthritis and autoimmune disorders. Despite its therapeutic efficacy, its clinical application is limited by adverse toxicological effects, particularly hepatic, renal, and reproductive toxicity [18]. Notably, TG has been shown to detrimentally affect sperm quality and testicular health [34,35,36]. In this study, a 4-week regimen of intragastric TG administration resulted in decreased body weight, compromised sperm quality, reduced levels of sex hormones, and damage to renal and hepatic tissues, ultimately leading to oligospermia in male rats. These outcomes corroborate findings from prior research [35,37]. Our investigation further revealed that intragastric TG administration significantly diminished sperm motility and concentration, suggesting the induction of spermatogenic arrest or apoptosis. TG is known to disrupt spermatogenesis by damaging testicular tissue, inhibiting spermatogonia proliferation, and inducing oxidative DNA damage [34,38]. Consistent with existing literature, the reductions in sperm metrics observed in the TG-treated group confirm the establishment of a robust reproductive toxicity model [34,35,36,39].

Optimal male fertility relies on the intricate integration and communication of various cell types within the testis [40]. Cytokines play a crucial role in regulating this complex process, which is often disrupted in the seminal plasma of hypo-fertile men. TNF-α is particularly significant as it stimulates the production of ROS, leading to sperm lipid peroxidation and apoptosis. Toxic substances can infiltrate the testis, promoting ROS production and initiating the mitogen-activated protein kinase (MAPK) cascade. This activation can trigger sperm apoptosis via the MAPK/PI3K/AKT signaling pathways, thereby disrupting testicular architecture, reducing testosterone synthesis, and diminishing sperm quality [41,42,43,44,45,46,47]. Our study demonstrated that TG exposure created a state of oxidative stress in the testes, characterized by elevated ROS and MDA levels and decreased total T-AOC and SOD levels. Aitken et al. have observed that such stress conditions activate apoptotic pathways, resulting in mitochondrial dysfunction and oxidative DNA damage, thereby making spermatozoa particularly susceptible [48]. Furthermore, our results revealed that there was a significant decrease in TG the levels of serum and intra-testicular T while increasing the levels of LH, FSH, ALT, AST, BUN and CRE in rat serum, highlighting its toxicity and resulting in testicular tissue damage and the establishment of oligospermia. Consequently, it is important to identify a therapeutic substance that can mitigate the toxicity of TG.

Considering that the primary mechanism of TG toxicity involves oxidative stress, we investigated the therapeutic potential of COU, a phytoestrogen with antioxidant properties, alongside LC as a positive control. The results indicated that LC treatment significantly improved sperm motility, concentration, and antioxidant status (SOD, T-AOC) while reducing ROS and MDA levels, consistent with its known role in protecting against gonadal toxicity [49,50,51,52,53]. While some studies suggest divergent effects of phytoestrogens on semen health [54,55], others report benefits [56]. Our findings demonstrate that COU treatment significantly ameliorated TG-induced toxicity, improving body weight, sperm quality, and antioxidant markers while normalizing liver and kidney function markers (ALT, AST, BUN, CRE). These results support the potential of COU as an effective ameliorative agent.

Notably, significant enhancements in hormonal levels were observed in COU-treated rats, with serum testosterone levels, which were reduced in the TG group, showing a significant increase in the TG + COU-M and TG + COU-H groups. Conversely, the LH and FSH levels, which were elevated in the TG group, were significantly reduced in the TG + COU-M and TG + COU-H groups, with the TG + COU-H group achieving the most significant results among the treatment groups compared with the TG group. A similar restoration of hormonal balance due to phytoestrogen efficacy was observed by Hafezi et al. [53], who investigated the ameliorative effects of resveratrol in rats with azoospermia.

Beyond serum hormones, intra-testicular testosterone (ITT) is a critical determinant of spermatogenesis, typically maintaining concentrations 30–40 times higher than peripheral blood [57,58,59]. Nurdiana et al. [60] reported a reduction in FSH and LH levels, accompanied by an increase in T levels, attributing these changes to alterations in the feedback mechanism between the testis and hypothalamus. Decreased FSH levels suggest hypothalamic impairment, whereas increased FSH levels indicate testicular impairment [61], leading to a decrease in negative feedback from T, resulting in decreased sperm count, vitality, and motility [62]. In our study, TG treatment caused a significant decline in ITT and a significant increase in intra-testicular FSH and LH. Notably, COU administration significantly restored ITT, intra-testicular FSH and intra-testicular LH levels. Existing literature indicates that while ITT can decrease without immediate effects, there is a critical threshold below which spermatogenesis is compromised [63,64]. The recovery of ITT in the COU-treated groups suggests that the compound preserves the androgen microenvironment necessary for germ cell maturation, a mechanism that serum T measurements alone might not fully capture [65].

To elucidate the underlying molecular mechanisms, we employed network pharmacology analysis through relevant databases (Table S1A,B). This analysis predicted 7 hub genes potentially associated with oligospermia, including AR, MAPK1, TP53, NFE2L2, HIF1A, ESR1 and GMNN. Gene Ontology (GO) analysis suggested that these targets might be involved in regulating hormonal responses and cell differentiation. Furthermore, KEGG pathway analysis identified significant pathways implicated in the oligospermia model of experimental rats. Among these pathways, apoptosis, TNF/TNFR1 signaling, MAPK (ERK), and PI3K/AKT pathways as key predicted modulators due to their strong association with oxidative stress, apoptosis spermatogenesis, and cellular life cycle regulation [33]. The TNF/TNFR1 signaling pathway is known to potentially stimulate ROS generation and apoptosis [66,67,68,69]. Additionally, the PI3K/AKT and ERK1/2 pathways play pivotal roles in regulating cell survival and proliferation [70,71,72]. The computational analysis implied that TG might induce toxicity by inhibiting these survival pathways, while COU could exert its protective effects by modulating them.

We utilized network pharmacology analysis to investigate how COU mitigates TG toxicity through relevant databases (Table S1A,B). This analysis revealed 7 hub genes associated with oligospermia in male rats. A PPI network comprising the nodes AR, MAPK1, TP53, NFE2L2, HIF1A, ESR1 and GMNN was created using the STRING database and Cytoscape software. Gene GO analysis suggested that these proteins may be involved in regulating sperm concentration and motility through mediating biological processes, including hormonal response, negative regulation of transcription via RNA polymerase, branching morphogenesis, and inhibition of epithelial cell differentiation. These putative dysfunctions are predicted to occur within the microdomain of sperm cellular components, affecting tumor necrosis factor receptor binding and functions exhibited by healthy sperm cells. Furthermore, KEGG pathway analysis identified significant pathways implicated in the oligospermia model of experimental rats. Among these pathways, apoptosis, TNF/TNFR1 signaling, MAPK (ERK), and PI3K/AKT pathways were selected due to their strong association with oxidative stress, apoptosis spermatogenesis, and cellular life cycle regulation [33].

To validate these predictive insights, we examined the protein expression of PI3K, AKT, and ERK1/2 via Western blotting. Consistent with the in silico predictions, TG treatment significantly downregulated the expression of ERK1/2, PI3K, and AKT1, a change associated with increased apoptosis and tissue damage. Importantly, COU treatment reversed this suppression, significantly upregulating these proteins. This suggests that COU promotes germ cell survival and testicular repair by activating the PI3K/AKT and ERK1/2 signaling cascades, thereby counteracting the oxidative and apoptotic effects of TG.

## 5. Conclusions

In conclusion, the present study demonstrates that TG successfully induced oligospermia in rats, causing oxidative damage to testicular tissues. TG exerted its toxicity by elevating the levels of ROS, MDA, FSH, LH, ALT, AST, BUN and CRE, and reducing levels of SOD, T-AOC, ITT, and T, as well as sperm motility and concentration. Histopathological experiments revealed significant indicators of testicular injury caused by TG toxicity. COU administration significantly attenuates TG -induced toxic effects in male rats. Its antioxidant properties, as evidenced by increased SOD activity and elevated T-AOC levels, resulting in a reduction in oxidative stress markers, including ROS and MDA. This attenuation of oxidative stress contributes to the amelioration of testicular injury, as indicated by the restoration of T, FSH, LH, ALT, AST, BUN and CRE levels.

Based on network pharmacology analysis, we predicted that the PI3K/AKT and MAPK signaling pathways might serve as potential underlying mechanisms for these effects. Subsequent Western blot analysis confirmed that COU treatment significantly upregulated the expression of key proteins (PI3K, AKT, and ERK1/2) within these pathways. These findings suggest that the antioxidant and ameliorative effects of COU may be associated with the modulation of the PI3K/AKT and MAPK signaling cascades.

Overall, this study provides experimental evidence supporting the potential of COU as a therapeutic agent for oligospermia and offers predictive insights into its molecular mechanism, which warrants further investigation.

## Figures and Tables

**Table 1:** Comparison of biochemical and physiological parameters among experimental groups (mean ± SD).

Group	n	ALT ± SD	AST ± SD	BUN ± SD	CRE ± SD
		(U·L^−1^)	(U·L^−1^)	(mmol·L^−1^)	(μmol·L^−1^)
Control	8	9.86 ± 0.93	11.56 ± 1.38	8.12 ± 1.24	43.91 ± 3.42
TG + LC	8	15.11 ± 0.94 **##	23.64 ± 1.87 **##	9.775 ± 1.73 **##	57.28 ± 4.94 **##
TG	8	22.53 ± 1.90 **	35.13 ± 1.88 **	20.2 ± 2.90 **	136.30 ± 5.01 **
TG + COU-L	8	16.61 ± 1.23 **##	37.74 ± 0.97 **	12.52 ± 1.98 **##	50.05 ± 3.78 **##
TG + COU-M	8	13.88 ± 0.94 **##	12.79 ± 0.19 *##	9.37 ± 1.70 ##	44.46 ± 2.96 ##
TG + COU-H	8	13.55 ± 0.67 **##	14.3 ± 0.48 **##	10.11 ± 1.98 *##	52.71 ± 2.19 **##

Note: Compared with the Control group, “*” *p* < 0.05; “**” *p* < 0.01; compared with the TG group, “##” *p* < 0.01.

**Table 2:** Node Table based on Cytoscape ranked by degree of centrality.

Rank	Name	Degree
1	AR	5
2	MAPK1	5
3	TP53	5
4	NFE2L2	5
5	HIF1A	5
6	ESR1	5

## Data Availability

The original contributions presented in this study are included in the article/Supplementary Material. Further inquiries can be directed to the corresponding authors.

## Supplementary Materials

The following supporting information can be downloaded at: https://www.mdpi.com/article/10.3390/vetsci13030224/s1, Figure S1: Original images of Western-blotting; Table S1: The databases and the list of some software used in the study.

## Abbreviations

The following abbreviations are used in this manuscript:

ALT	alanine aminotransferase
AR	androgen receptor
AST	Aspartate aminotransferase
AKT	Protein kinase B
BCL2	B-Cell Lymphoma 2
BUN	Blood urea nitrogen
CASP3	Caspase 3
CAT	Catalase
CCNB1	Cyclin B1
CDK2	Cyclin-Dependent Kinase 2
COU	Coumestrol
CRE	Creatinine
ESR1	Estrogen Receptor 1
FSH	Follicle-stimulating hormone
GO	Gene Ontology
GPX	Glutathione peroxidase
HSP90AA1	Heat Shock Protein 90 Alpha Family Class A Member 1
ITT	Intra-testicular Testosterone
LC	Levo Carnitine
LCs	Leydig cells
LH	Luteinizing hormone
MAPK1/ERK2	Mitogen-Activated Protein Kinase 1/Extracellular Signal-Regulated Kinase 2
MDA	Malondialdehyde
PARP1	Poly (ADP-Ribose) Polymerase 1
PI3K	Phosphoinositide 3-kinase
RIP1	Receptor-Interacting Protein 1
ROS	Reactive oxygen species
SOD	superoxide dismutase
T	Testosterone
T-AOC	Total antioxidant capacity
TCM	Traditional Chinese Medicine
TG	Tripterygium glycosides
TNF	Tumor Necrosis Factor
TRADD	TNF Receptor-Associated Death Domain
TRAF2	TNF Receptor-Associated Factor 2
