# Supplementary material for: Evaluating the Antioxidant Potential of Coumestrol in the Treatment of Tripterygium Glycoside-Induced Oligospermia in Rats and Its Potential Mechanisms
Source: Vet Sci. 2026 Feb 26;13(3):224. doi: 10.3390/vetsci13030224 (PMC13030545; doi:10.3390/vetsci13030224)
Supplement: Supplementary file 1 [file vetsci-13-00224-s001.zip › vetsci-4131835-supplementary,table S1.pdf]

Evaluating the Antioxidant Potential of Coumestrol in the Treatment of Tripterygium Glycoside-Induced Oligospermia in Rats and Its Potential Mechanisms

Yongzheng Liu[1], Sikai Chen[1], Kang An[1], Long Chen[1], God's power Bello-Onaghise[1,2], Yu Zhang[1], Shunda Li[1], Mo Chen[3], Haoran Wang[4], Qianwei Qu[1*], and Yanhua Li[1*]

[1]College of Veterinary Medicine, Northeast Agricultural University, 600 Changjiang Road, Xiangfang, Harbin, Heilongjiang 150030, P. R. China.

[2]Department of Animal Science, Faculty of Agriculture, University of Benin, Benin City, Nigeria.

[3]State Key Laboratory of Veterinary Biotechnology, Harbin Veterinary Research Institute, Chinese Academy of Agricultural Sciences, Harbin, 150069, China.

[4]Southern Medical University, 1023 Shatainan Road, Guangzhou, Guangdong Province, China

***Corresponding author:** Yanhua Li, **Email:** liyanhua@neau.edu.cn; Qianwei Qu, **Email:** qwqu@neau.edu.cn

Tables S1: The databases and the list of some software used in the study

Table S1 A: Databases for Network Pharmacological Analysis

S/N	Database	Function	Website
1	PubMed	Curating related articles	https://pubmed.ncbi.nlm.nih.gov/
2	Traditional Chinese Medicine System Pharmacopeia (TCMSP)	Identification of Bioactive Compounds from Chinese Medicine	https://old.tcmsp-e.com/load_intro.php?id=27
3	PubChem	2D structure and SMILES of Bioactive compounds	https://pubchem.ncbi.nlm.nih.gov
4	SWISS ADME	For Drug likeness of bioactive compounds	http://www.swissadme.ch/index.php
5	DrugBank	Bioactive Targets and Pathways	https://go.drugbank.com
6	UniProt	For validating the target gene ID	https://www.uniprot.org
7	DisGeNet	For disease-related genes	https://www.disgenet.org
8	GeneCards	For disease-related genes	https://www.genecards.org
9	MalaCards	MalaCards	malacards.org
10	STRING	Protein-protein interaction	https://string-db.org
11	ChEMBL	Identification of potential bioactive target	https://www.ebi.ac.uk/chembl
12	STITCH	Identification of potential bioactive target	http://stitch.embl.de/
13	ShinyGO 0.81	For performing Gene Ontology (GO) and Kyoto Encyclopedia of Genes and Genomes (KEGG) enrichment analyses	http://bioinformatics.sdstate.edu/go/

Table S1B: Software for Network Pharmacological Analysis

S/N	Software	Function	Version
1	Cytoscape	Network visualization	3.10.1
2	CytoHubba	Hub genes network generation (Plugin in Cytoscape)	3.10.1
3	Microsoft Excel	Organization of Data	2024
4	Venny2.1	For drawing up the venin diagram to show common genes between bioactive and diseases	2.1
